# Management of Post-Colonoscopy Syndrome with a Nutraceutical Intervention Based on *Hericium erinaceus*: A Retrospective Two-Arm Multicentre Analysis

**DOI:** 10.3390/nu17193152

**Published:** 2025-10-02

**Authors:** Antonio Tursi, Alessandro D’Avino, Giovanni Brandimarte, Giammarco Mocci, Raffaele Pellegrino, Alessandro Federico, Edoardo Vincenzo Savarino, Antonietta Gerarda Gravina, the HERICIUM-COLON Study Group

**Affiliations:** 1 Territorial Gastroenterology Service, Barletta-Andria-Trani Local Health Agency, Via Fornaci, 76123 Andria, Italy; 2 Department of Medical and Surgical Sciences, Catholic University of Rome, Largo A. Gemelli, 00168 Roma, Italy; 3 Department of Internal Medicine, IRCCS Istituto Dermopatico dell’Immacolata, 00167 Roma, Italy; 4 Consorzio Universitario Humanitas, Via della Conciliazione, 00193 Roma, Italy; 5 SC Gastroenterologia, ARNAS Brotzu, Piazzale Alessandro Ricchi, 09047 Cagliari, Italy; 6 Hepatogastroenterology Division, Department of Precision Medicine, University of Campania Luigi Vanvitelli, Via Luigi de Crecchio, 80138 Napoli, Italy; 7 Gastroenterology Unit, Department of Surgery, Oncology and Gastroenterology, University of Padova, Via VIII Febbraio, 35121 Padova, Italy

**Keywords:** post-colonoscopy syndrome, nutraceutical supplementation, *Hericium erinaceus*, butyrate, probiotics, *Lactobacillus acidophilus*, *Bifidobacterium animalis*, *Lactiplantibacillus plantarum*

## Abstract

**Background**: Post-colonoscopy syndrome is an emerging clinical entity characterised by the onset of gastrointestinal symptoms following a colonoscopy. The current management of this syndrome has not yet been established, although probiotics have been proposed. The therapeutic potential of a combination nutraceutical compound based on HBQ-Complex^®^, butyrate, and probiotics (*Lactobacillus acidophilus*, *Bifidobacterium animalis*, and *Lactiplantibacillus plantarum*) in this setting remains unknown. **Methods**: A retrospective, multicentre, observational study was conducted in adult patients undergoing colonoscopy in the absence of known gastrointestinal diseases, assessing the onset of upper and lower gastrointestinal symptoms post-colonoscopy immediately after the procedure (T_0_), at 2 weeks (T_1_), and 4 weeks (T_2_) thereafter, using a VAS (0–10). Two groups were analysed, one undergoing nutraceutical supplementation and a control group. **Results**: A total of 599 patients were included (64.9% receiving nutraceutical supplementation and 35% in the control group). Several variations were observed involving the treated group compared to the control for abdominal pain (59.9% vs. 33.3%), meteorism (64.9% vs. 35.1%), diarrhoea (46.9% vs. 19.5%), and bloating (59.3% vs. 26.7%) (*p* < 0.001 for all). Logistic regression analysis showed a reduction in constipation (OR: 3.344) and bloating (OR: 3.791) scores. **Conclusions**: Nutraceutical supplementation based on this combinational compound was associated with a reduction in gastrointestinal symptoms arising after colonoscopy, suggesting potential benefit in this setting. These findings pose a rationale for controlled prospective studies to confirm such evidence in broader clinical settings.

## 1. Introduction

Colonoscopy is a cornerstone endoscopic diagnostic procedure for diagnosing and treating colorectal and ileal segment pathologies [1]. A fundamental prerequisite for this examination is the patient’s appropriate and effective bowel preparation, aimed at inducing sufficient evacuations to cleanse the colon, thereby enabling a clear endoscopic view unimpeded by the presence of faecal residues [2], especially in conditions where the macroscopic characteristics of the mucosa must be optimally appreciated [3]. However, current evidence suggests the possible de novo onset of gastrointestinal symptoms following colonoscopy [4], collectively and non-specifically defined as *post-colonoscopy syndrome*.

This entity, although not nosologically codified [5,6,7,8], thus represents a clustering of potential gastrointestinal symptoms arising after colonoscopy and not attributable to any other defined clinicopathological condition.

Various pathophysiological hypotheses have been proposed relating to the impact of bowel preparation on the colonic micro- and macroenvironment [9]. Among them, bowel preparation-induced alterations in the qualitative and quantitative balance of the gut microbiota (induced dysbiosis) and depletion of the mucus layer covering the luminal surface of colonocytes have been suggested [9,10]. Previous evidence on the development of post-colonoscopy gastrointestinal symptoms or syndromes is particularly scarce. In a propensity score analysis conducted by Vejravelu et al. [11] on over four hundred thousand patients undergoing screening colonoscopy, a certain risk of irritable bowel syndrome following colonoscopy was identified in cases with a history of prior antibiotic use. Lee et al. [12], on the other hand, developed a logistic regression model to determine variables associated with abdominal pain after colonoscopy in a prospective study including one thousand screening colonoscopy patients, identifying colonoscopy duration, female sex, prior irritable bowel syndrome, and conscious sedation as predictive factors.

Several studies have already examined the therapeutic potential of probiotic supplementation [13] in improving post-colonoscopy gastrointestinal symptoms. Although meta-analytical analysis of these studies indicates a potential benefit, the effects fail to achieve marked statistical significance, highlighting the need for more research on the subject [14]. In particular, the evidence related to other biotics, such as postbiotics [15] and prebiotics [15], as well as nutraceuticals [16] for this specific indication, is even more limited.

Among the nutraceutical compounds used in real-life clinical practice in Italy as dietary supplements, a freely available combination product (that is, without the requirement for a medical prescription) based on HBQ-Complex^®^ is derived from the *Hericium erinaceus* (HE) mushroom, berberine, and quercetin. Several studies have demonstrated preclinical anti-inflammatory potential of HE in various gastrointestinal tract disorders, especially inflammatory bowel diseases [17]. Although limited by the absence of randomised controlled trials specifically evaluating this combination (i.e., HBQ-Complex^®^) in the context of gastrointestinal indications, some evidence is available from preliminary studies. In detail, HBQ-Complex^®^ has been shown, in real-world practice studies, to potentially increase clinical remission rates in symptomatic uncomplicated diverticular disease [18] and in mild-to-moderate ulcerative colitis, in combination with 5-aminosalicylic acid derivatives [19]. In the context of inflammatory bowel diseases, a preclinical study has also shown the potential of HBQ-Complex^®^ to reduce the inflammatory burden (in terms of inflammatory mediators such as cyclooxygenase-2 and tumour necrosis factor) in an ex vivo biopsy model incubated with the compound in both ulcerative colitis and Crohn’s disease [20].

Currently, there are no specific recommendations or guidelines from international digestive endoscopy societies regarding the medical management of post-colonoscopy syndrome, nor is there any evidence supporting supplementation with the aforementioned combination product in this setting.

## 2. Materials and Methods

### 2.1. Study Design

The design of this study is observational, multicentre, real-life, and structured into two analytical arms. The study was conducted through a strictly anonymous retrospective collection of data related to post-colonoscopy visits for the onset of gastrointestinal symptoms recorded over the past one year (from 2024 to 2025). The two study arms were differentiated based on the use or non-use of the investigated nutraceutical intervention. The study has been drafted following the Strengthening the Reporting of Observational Studies in Epidemiology (STROBE) checklist for retrospective observational studies. No missing data are expected, as patients were only included when all relevant variables of interest were fully collected.

The study was conducted in compliance with the Declaration of Helsinki [21], and data were collected in complete anonymity, with no nominal information included in the study records. Given the study’s fully anonymous nature, which prevents any possibility of identifying patients from the collected data and following the Italian Medicines Agency determination of Mar 20, 2008, approval from the institutional review board was exempted due to its observational and entirely anonymous design.

### 2.2. Inclusion and Exclusion Criteria

Both study groups included patients aged between 18 and 75 years who had no symptoms related to abdominal pain, epigastric pain, bloating or meteorism, dyspepsia, diarrhoea, or constipation prior to undergoing a colonoscopy for any indication (e.g., colorectal cancer screening, family history of colorectal neoplastic diseases, haematochezia, or rectal bleeding). We excluded from the analysis patients who had undergone previous surgical interventions involving the gastrointestinal tract, except for an uncomplicated appendectomy. Additionally, we excluded patients with a known diagnosis of colorectal cancer or other organic or functional gastrointestinal disorders (according to the ROME IV criteria [22]) that could potentially be the source of the previously mentioned gastrointestinal symptoms. Furthermore, we excluded pregnant or breastfeeding patients at the time of the colonoscopy and those who underwent biopsy sampling or operative resection procedures (e.g., polypectomy, mucosectomy, submucosal dissection) during the colonoscopy. Finally, patients with psychiatric disorders or other conditions that could potentially cause gastrointestinal symptoms (e.g., uncontrolled diabetes, thyroid or adrenal disorders) were excluded from data collection.

The division between the two study groups was based on the use of the combination compound described in Section 2.3 at the standard dosage of one tablet per day for four weeks following the colonoscopy in the first group (nutraceutical supplementation group). Conversely, patients were included in the analysis as part of the control group if they did not receive the nutraceutical intervention.

The prescription of nutraceutical supplementation (and thus indirectly the allocation to the groups) was determined according to clinical practice, based on the physician’s case-by-case assessment, and was neither decided *a priori* nor the result of randomisation, given the observational and real-life design of the study.

### 2.3. Investigated Nutraceutical Combination Compound

The compound under evaluation is freely marketed in Italy and registered in the database of food supplements notified to the Ministry of Health under code 168,374 (Enteroflegin Flora^®^, Fenix Pharma SOC.COOP.P.A, Rome, Italy). Fenix Pharma was not involved in the designing, conducting, interpretating, or writing the study. The main characteristics of this compound are outlined in Table 1. The combination of HE, quercetin, and berberine is defined as HBQ-Complex^®^ and is protected by a patent (patent number 102021000006581, filed on 18 March 2021 with the Italian Patent and Trademark Office, Division XIV, and granted on 5 April 2023).

In detail, the ingredients of this compound are as follows: HBQ-Complex^®^-HE [*Hericium erinaceus* (Bull.) Pers., sporophore] powder, titrated to 5% polysaccharides, HE [*Hericium erinaceus* (Bull.) Pers., sporophore] extract, titrated to 30% polysaccharides, quercetin 98%, berberis (*Berberis vulgaris* L., root bark) extract, titrated to 97% berberine, sodium butyrate, capsule shell (coating agent: hydroxypropyl methylcellulose; stabiliser: gellan gum); bulking agents: dicalcium phosphate, microcrystalline cellulose; *Lactobacillus acidophilus* SGL11 100 Bn CFU/g, *Bifidobacterium animalis* subsp. lactis SGB06 50 Bn CFU/g, niacin (nicotinamide); anti-caking agents: magnesium salts of fatty acids, silicon dioxide (nano); *Lactiplantibacillus plantarum* SGL07 100 Bn CFU/g, gastro-resistant coating (water; coating agents: ethyl cellulose, ammonium hydroxide; emulsifier: mono- and diglycerides of fatty acids; anti-caking agents: sodium carboxymethylcellulose, talc; stabiliser: polydextrose), biotin (D-biotin). Further and more detailed information on the probiotics included in the product is presented in Tables S1–S3.

### 2.4. Collected Variables and Data Collection Timeline

The following baseline variables were extracted from the analysed endoscopic reports: sex, age, comorbidities, concomitant therapies and previous surgical interventions, ongoing medical treatments, smoking status, and the type of dietary regimen reported by the patient. Additionally, colonoscopy-specific variables were assessed, including the indication for colonoscopy, the type of bowel preparation performed, the number of days of a fibre-free diet followed before the colonoscopy, the level of bowel preparation according to the Boston Bowel Preparation Scale (BBPS) [23], and, if applicable, the Diverticular Complication and Inflammation Assessment (DICA) [24] classification for colonic diverticula.

Furthermore, since the standard management practice of the included centres also evaluated an 11-level VAS Likert scale [25] (ranging from 0 to 10) for the severity of gastrointestinal symptoms (those already listed in the inclusion criteria) at baseline early after the colonoscopy (T_0_), at 2 weeks (T_1_), and 4 weeks (T_2_) after the colonoscopy, these data were also collected.

### 2.5. Study Outcomes

The primary outcome was the evaluation of improvement in gastrointestinal symptoms (one or more among abdominal pain, epigastric pain, meteorism, bloating, dyspepsia, diarrhoea, and constipation) that newly emerged post-colonoscopy and were not attributable to any other gastrointestinal pathology at T_2_, defined as a 50% reduction in the VAS score compared to T_0_. This assessment was also conducted at T_1_ compared to T_0_ as a secondary outcome. In addition, an additional secondary outcome was the safety of the investigated nutraceutical compound. Finally, as the last exploratory secondary outcome, the variation in study intervals (i.e., T_0_–T_1_, T_0_–T_2_) of the total VAS score (defined as the sum of the individual VASs for each symptom) was assessed, as well as the proportion of patients who, over the same intervals, experienced a 50% reduction in the total VAS score.

### 2.6. Statistical Analysis

Descriptive statistics were used for data presentation. Continuous variables were presented as the mean ± standard deviation or median with the corresponding interquartile range. The choice between these methods depended directly on the distribution of the continuous variable (normal or non-normal), which was preliminarily assessed using the Kolmogorov–Smirnov test. The variation in VAS scores over time was assessed using the Wilcoxon signed-rank test, while differences in outcome rates were analysed using the Chi-square test. A statistical significance threshold of *p*-value < 0.05 (strictly two-tailed) was adopted, setting the alpha error at 0.05. To examine the differences in VAS variations, differential mean variables (Δ) were identified for each of the gastrointestinal symptoms assessed. Further analysis of the study outcomes will aim to identify variables acting as predictors of a 50% improvement in the VAS at T_2_ compared to baseline (T_0_) for each assessed gastrointestinal symptom. This will be considered a dichotomous dependent variable for the appropriate logistic regression analyses, with nutraceutical supplementation intake evaluated as an independent variable to assess the magnitude of each improvement over the considered time interval. The regression model will be assessed for goodness-of-fit using the Hosmer-Lemeshow test and the Cox and Snell R^2^ and Nagelkerke R^2^ values. Results will be expressed as the exponential value of B, i.e., exp (B). This will be presented as the Odds Ratio (OR), with the risk measure expressed as OR and the corresponding 95% confidence interval. The ORs will be adjusted for clinically relevant confounding variables (aOR).

Statistical analyses were performed using IBM^®^ SPSS^®^ software (version 25, IBM Corp.^©^, Armonk, NY, USA), while graphical representations were generated using GraphPad PRISM^®^ software (version 9.5.0, GraphPad Software LLC^©^, Boston, MA, USA). Sample size calculations were conducted using G*Power software (version 3.1.9.6, Faul, Erdfelder, Lang, & Buchner, Düsseldorf, Germany). The sample size calculation was based on the comparison of two independent proportions concerning the primary endpoint (proportion of “responders”, i.e., patients with a ≥ 50% reduction in VAS at T_2_ compared with T_0_), assuming a two-tailed test, α = 0.05, and power (1–β) 0.80. Based on a clinically relevant minimum difference of 15 percentage points (p_treatment_ = 0.45 vs. p_control_ = 0.30), the corresponding effect size (Cohen’s h) was 0.31. This yielded a minimum sample size of approximately 162 subjects per arm (total 324) with 1:1 group allocation.

## 3. Results

### 3.1. Baseline Characteristics of the Sample

**Figure 1:**
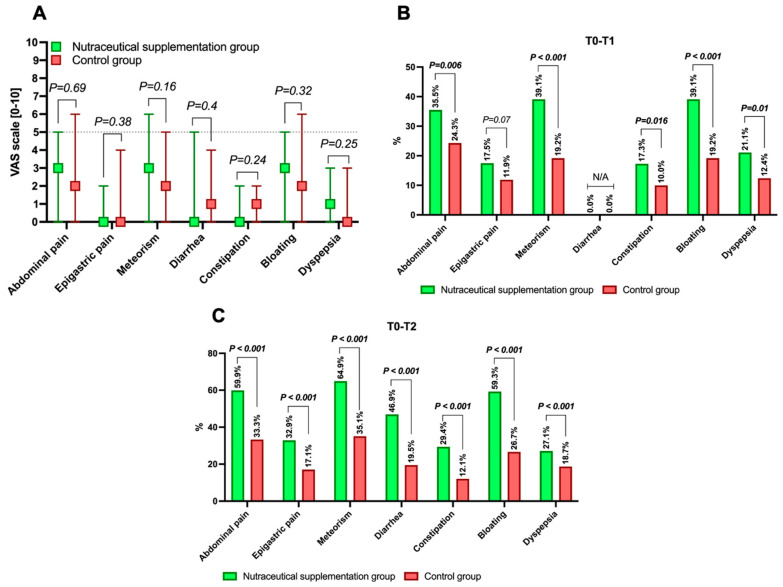
Baseline levels of gastrointestinal symptoms measured using the VAS (0–10) in the population undergoing nutraceutical supplementation post-colonoscopy and in the control group (**A**), as well as the study outcomes (**B**,**C**). Continuous variables are expressed as median and interquartile range. The *p*-value presented is calculated with an alpha error of 5% (*p* < 0.05) for significance, assessing whether differences exist for the specified parameter between the two groups. *p*-values < 0.05 are presented in bold for a clearer definition. The outcome of the response on the VAS (with a 50% reduction) compared to the baseline is defined at both time points T_1_ (**B**) and T_2_ (**C**). In the case of insufficient sample size to perform a comparison, the analysis is indicated as not applicable (N/A).

A total of 599 patients were included in the analysis, with 389 (64.9%) in the nutraceutical supplementation group and 210 (35%) in the control group. The included patients resulted from the exclusion of 274 individuals (from an initial screened pool of 873 patients) who had missing data and/or did not meet the inclusion criteria. The median age of the entire sample was 57.3 (47.2–65.2), with a predominance of females (309, 51.5%). The primary indication for colonoscopy was colorectal cancer screening (234, 39.06%), and the most commonly used bowel preparation was 2 L PEG plus citrate/simethicone (172, 28.71%). Overall, the sample exhibited a good level of bowel preparation, identifying a rate of at least adequate bowel preparation of 89.1%. Regarding diverticulosis, present in 41% (246/599) of the entire sample, most of the sample had an uncomplicated one with a DICA 1 classification (163/599, 27.21%). As shown in Table 2, the two study groups demonstrated a reasonable degree of homogeneity, except for metabolic comorbidities, since they were found in a higher percentage in patients undergoing nutraceutical supplementation compared to control patients (32.9% vs. 23.3%, *p* = 0.015). Figure 1A also demonstrates homogeneity in VAS scores between the two groups at T_0_.

### 3.2. Study Outcomes: Results

The study outcomes were achieved by a substantial portion of the sample, as shown in Figure 1B,C. Concerning the primary outcome (Figure 1C), all symptoms improved more significantly in terms of outcome achievement rates in the nutraceutical supplementation group compared to the control group, including abdominal pain (59.9% vs. 33.3%, *p* < 0.001), epigastric pain (32.9% vs. 17.1%, *p* < 0.001), meteorism (64.9% vs. 35.1%, *p* < 0.001), diarrhoea (46.9% vs. 19.5%, *p* < 0.001), constipation (24.9% vs. 12.1%, *p* < 0.001), bloating (59.3% vs. 26.7%, *p* < 0.001), and dyspepsia (27.1% vs. 18.7%, *p* < 0.001). As evident from the percentages, the symptoms most impacted by supplementation, achieving the highest improvement rates, were abdominal pain, meteorism, diarrhoea, and bloating (Figure 1C).

For the secondary outcome (Figure 1B), improvement was also observed, albeit with predictably lower rates than the primary outcome, except for epigastric pain, where there was a uniform response regardless of nutraceutical supplementation (15.5% in the nutraceutical group vs. 11.9% in the control group, *p* = 0.07, Figure 1B).

### 3.3. VAS Scores Variations over Study Times

**Figure 2:**
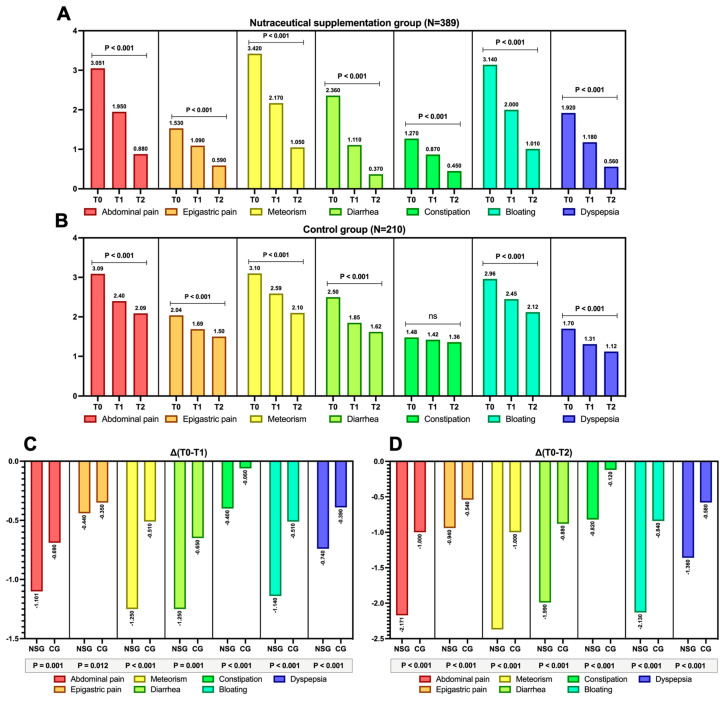
Variation in the means of each gastrointestinal symptom assessed using the VAS (0–10) both at baseline (T_0_) and at T1 and T2 in the group undergoing nutraceutical supplementation (NSG) post-colonoscopy (**A**) and in the control group (CG), as shown in panel (**B**). Continuous variables are represented as means. The *p*-value tests whether the variations (T_0_–T_1_, T_0_–T_2_) are significant, with an alpha error of 5% (*p* < 0.05) for significance. Significant data (*p* < 0.05) are boldly highlighted for easier reference. *p*-values > 0.05 are indicated as “ns”, meaning not significant. The overall sample size (N) is provided for each group. Additionally, the mean differences (Δ) are expressed for both the T_0_–T_1_ interval (**C**) and the T_0_–T_2_ interval (**D**) for the NSG and CGs. In this case, the *p*-value shown highlights the difference in differential trends (Δ) between the two groups.

Gastrointestinal symptoms assessed using the VAS demonstrated a reduction trend over time compared to baseline at both T_1_ and T_2_, significantly in both groups (Figure 2A,B), except for constipation in the control group, where no variation was observed (Figure 2B, *p* > 0.05). However, when evaluating the magnitude of this variation, it is evident that the differential mean trends for both ΔT_0_–T_1_ and ΔT_0_–T_2_ show a markedly more pronounced improvement for each gastrointestinal symptom in the nutraceutical supplementation group compared to the control groups (Figure 2C,D, *p*-value for all < 0.001).

**Figure 3:**
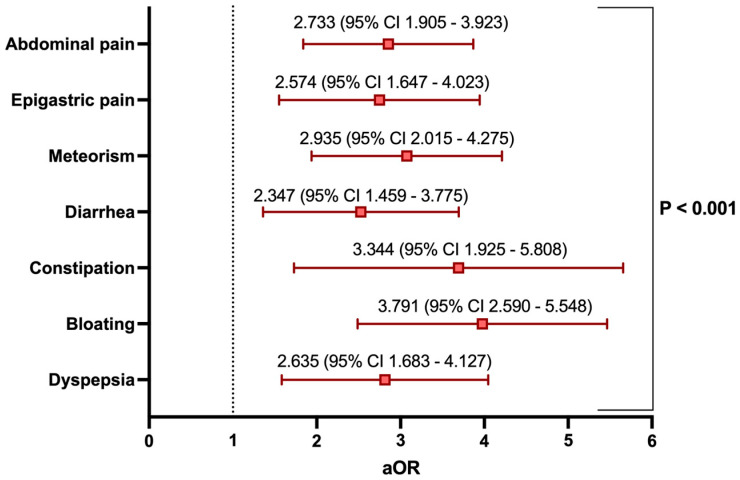
Logistic regression analysis on the impact of nutraceutical supplementation on the improvement of at least 50% in the VAS (0–10) of gastrointestinal symptoms at T2 compared to baseline (T_0_). Odds ratios (OR) and their corresponding 95% confidence intervals (95% CI) are presented. The ORs are adjusted (aOR) since they have been corrected for age, sex, alcohol consumption, smoking, examination indication, usual diet, pre-colonoscopy diet duration, and degree of bowel preparation. An aOR > 1 corresponds to an increased likelihood of achieving a 50% improvement in the respective parameter. Each of the aORs calculated in the regression demonstrated statistical significance with an alpha error of 5% (*p* < 0.05).

Additionally, to quantify the impact of the nutraceutical supplementation on the reduction in each assessed gastrointestinal symptom concerning the primary outcome (i.e., a 50% improvement in the VAS at T_2_), logistic regression identified an improving risk trend for each symptom. However, the most pronounced improvement was observed for constipation (OR: 3.344) and bloating (OR: 3.791), as shown in Figure 3 (*p* < 0.001).

**Figure A1:**
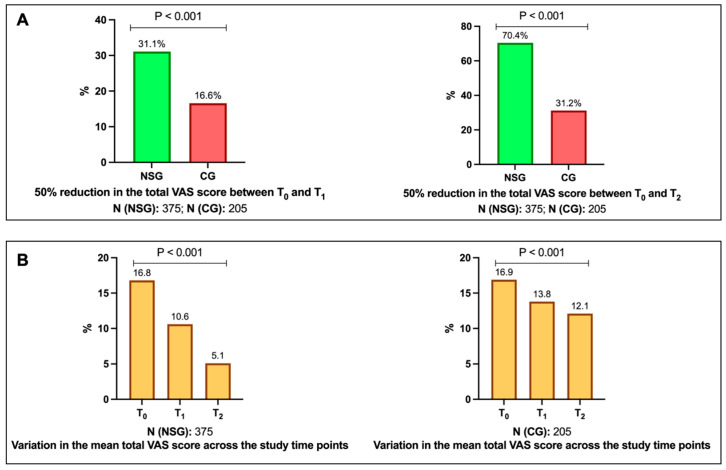
Outcome of a 50% reduction in the total visual analogue scale (VAS) score, derived from the sum of the individual VAS scores for each symptom, across the different study time points, namely T_0_, T_1_, and T_2_ (**A**), both in the nutraceutical supplementation group (NSG) and in the control group (CG), with the sample size indicated for each comparison (N). The variations in the mean total VAS score at the three study time points are also shown for both groups (**B**). The *p*-value in panel (**A**) refers to the difference in rates between the two groups, whereas in panel (**B**) it refers to the change in the total VAS score over time.

Figure A1A depicts the variation in the total VAS score (cumulative sum of the VAS scores for each symptom) in both groups, showing, indeed, a significant change in both cases, although with greater negative variations in the group receiving the nutraceutical compound (*p* < 0.001 for both groups). Finally, as shown in Figure A1B, in support of the above findings, 31.1% and 70.4% of patients receiving the compound experienced a 50% reduction in the total VAS score at the T_0_–T_1_ and T_0_–T_2_ intervals, respectively, compared with 16.6% and 31.2% in the control group (*p* < 0.001).

## 4. Discussion

This multicentric analysis demonstrates that the evaluated nutraceutical combination compound has the potential to positively affect a broad spectrum of emerging post-colonoscopy symptoms, as the primary outcome in our setting was achieved to a greater extent in the nutraceutical supplementation group (Figure 2).

Although an invasive technique, colonoscopy is relatively safe in experienced hands, particularly in diagnostic settings, with a reported perforation rate of approximately 0.005–0.085% and post-colonoscopy bleeding in about 0.001–0.687% of cases [26]. Beyond these more serious complications, the post-colonoscopy syndrome, as previously mentioned, lacks a substantial body of evidence, partly because some studies have focused explicitly on abdominal pain, particularly in the immediate post-procedural period, where certain contributing factors have been identified, including the duration of the colonoscopy and female sex [12]. However, fewer data are available regarding longer-term follow-up after colonoscopy. In response to this syndrome, data on probiotic supplementation are available in the literature. Muzellina et al. [14], as already stated, conducted a meta-analytical review of data from ten studies, predominantly using *Bifidobacterium* or *Lactobacillus* strains. Among these, seven studies specifically assessed probiotics’ effects post-colonoscopy, demonstrating improvements in symptoms such as bloating, abdominal pain, nausea, and vomiting, although statistical significance was not achieved in the examined studies.

Among the most comprehensive studies available, Hung et al. [27] examined the potential of a probiotic containing *Lactobacillus casei* spp. *rhamnosus* GG by randomising nearly three hundred patients to receive it either one week before and after colonoscopy, only one week before or after, or not at all. Symptoms were assessed using the Gastrointestinal Symptom Rating Scale [28], highlighting that abdominal pain and indigestion-related symptoms significantly improved in all intervention groups except for the untreated patients. Additionally, Bonavina et al. [29], in one of the largest quasi-experimental studies conducted on nearly three thousand patients, evaluated an intervention based on a combination probiotic (*Lactiplantibacillus plantarum* LP01 [30,31,32], *Lactobacillus lactis* subspecies *cremoris* LLC02 [33,34], and *Lactobacillus delbrueckii* LDD01 [35,36,37,38]) administered for four weeks post-colonoscopy. The mean age of the patients was 56 years, thus comparable to our study, and the symptoms analysed were mainly related to the lower gastrointestinal tract (abdominal pain, bloating, flatulence, and borborygmi). Their findings highlighted that, at baseline, immediately after the colonoscopy, abdominal pain and bloating were the most prevalent symptoms, which markedly decreased in both frequency and intensity after four weeks of treatment. However, the authors did not *a priori* exclude patients with pre-colonoscopy gastrointestinal symptoms, thereby limiting the comparison with regard to a strict clustering of post-colonoscopy syndrome.

In addition, these data are difficult to compare with our findings (except for the probiotic component), as probiotics were only one constituent of the nutraceutical compound assessed in this investigation. In any case, excluding probiotics and considering only HBQ-Complex^®^, the data on the improvement of gastrointestinal symptoms in patients with symptomatic uncomplicated diverticular disease [18] and on bowel movement frequency and rectal bleeding in patients with mild-to-moderate ulcerative colitis [19] suggest that this component may still play a role in symptom improvement within this setting. Additionally, since bowel preparation and colonoscopy can induce microscopic inflammatory changes [9,10], it is known that HBQ-Complex^®^, in an ex vivo setting, can reduce levels of pro-inflammatory cytokines such as tumour necrosis factor and promote the production of interleukin-10 within the intestinal mucosal microenvironment [20]. However, these remain broad hypotheses, limited by the fact that they have not been specifically evaluated in the setting of the post-colonoscopy syndrome and by the incomplete understanding of the underlying pathophysiological mechanisms of this condition.

Although our data suggest an improving trend for all assessed symptoms in the post-colonoscopy phase, some of these experienced a more pronounced improvement, particularly symptoms related to bowel gas (i.e., bloating and meteorism) as well as those associated with altered bowel habits (Figure 2 and Figure 3). It is indeed known that a positive modulation of the gut microbiota, that is, one which discourages the selection of pathobionts while promoting the growth of a health-promoting flora, can be achieved through the selection of bacteria producing short-chain fatty acids (SCFA). This is because such SCFA (particularly butyrate, which is also present in this nutraceutical supplementation) strengthen the epithelial barrier by improving intestinal permeability (positively inducing proteins such as occludins, zonulins, and claudins), inducing the production of glycoprotein mucins that protect the mucosa, stimulating the production of antimicrobial peptides and beta-defensins, and promoting the differentiation of T cells into regulatory T cells (through the promotion of Foxp3 by butyrate) [39].

Clearly, this compound, containing three bacteria with scientific evidence supporting their probiotic properties [40,41,42], already suggests the mechanism underlying these beneficial effects. However, the other elements of this nutraceutical compound also have evidence supporting improvements in gut microbiota. In detail, HE administered as a dry powder in a study conducted on healthy volunteers without known gastroenteric pathologies (as is indeed the case with our sample) led to an increase in SCFA-producing bacteria, enhancing the alpha diversity of the microbiota (specifically, strains of *Bifidobacterium* and *Bacteroides*, as well as other SCFA-producing bacteria) [43]. It is known that other polysaccharides from the HE mycelium have similar functions [17]. Furthermore, within the components of the studied combination compound, niacin has been shown to promote the selection of SCFA-producing bacteria through the activation of the butyrate receptor GPR109A, expressed on enterocytes, macrophages, and antigen-presenting dendritic cells [44]. By binding to this receptor, niacin promotes the production of anti-inflammatory cytokines such as interleukin-10 and supports the differentiation of gut regulatory T cells [44]. Similar beneficial properties for the gut microbiota have also been described for berberine [45] and quercetin [46].

This clearly warrants an important clarification: since this work assessed real-life effectiveness, all the considerations outlined above are speculative hypotheses (based on properties/evidence demonstrated in similar or preclinical settings), which call for caution regarding mechanistic aspects and require specifically designed studies to address this purpose.

This study has some limitations. It is a non-controlled analysis, which carries a certain risk of selection and recall biases, although the large sample size and the multicentric nature of the study partially mitigate these. Given the observational and real-world nature of the study [47,48,49,50,51], no randomised allocation of patients to each group was carried out. In addition, as this is a real-life study, the absence of a placebo arm and the lack of mechanistic considerations limit the inferences that can be drawn regarding the causal relationships of nutraceutical supplementation.

Certainly, the VAS as an evaluative tool was the result of a pragmatic choice, since it is not *de iure* standardised for assessment in the clinical setting under investigation in the present study. Nonetheless, the VAS has been widely employed in gastroenterology to gauge the symptomatic severity of gastrointestinal symptoms. For instance, Tsuda et al. [52] assessed the symptoms of patients with ulcerative colitis using an 11-point VAS (0–10) and suggested its correlation with the endoscopic activity of the disease. Similarly, Bengtsson et al. [53] employed a VAS in a clinical trial to evaluate the psychological well-being of patients with irritable bowel syndrome (IBS). Moreover, Bengtsson et al. [54] also used a specifically designed VAS (VAS-IBS) to assess the symptoms of this functional gastrointestinal disorder. Finally, moving beyond the realm of lower gastrointestinal disorders, the VAS has also been widely employed for the assessment of upper gastrointestinal symptoms [55,56].

In addition to the VAS, laboratory parameters (e.g., markers of intestinal inflammation such as faecal calprotectin or additionally microbiome analysis) were not assessed, nor were in-depth histological evaluations performed procedures that would have been challenging to implement in an observational setting. Since this is not a randomised controlled trial, a diary-based control of therapeutic adherence was not ensured for all patients. Therefore, these considerations highlight the imperative need to re-evaluate this nutraceutical compound in a prospective setting through the design of a randomised controlled trial, especially considering the promising data obtained in this analysis with a substantial sample size.

## 5. Conclusions

In conclusion, these data suggest that a short 4-week intervention with the nutraceutical combination described in this study was associated with an improvement in de novo post-colonoscopy gastrointestinal symptoms compared to patients who did not receive the intervention.

## Figures and Tables

**Table 1:** Composition of the nutraceutical combination compound supplemented in the retrospective analysis for managing post-colonoscopy syndrome.

Mean Values	In One Capsule	%RNV ^1^	In Two Capsules	%RNV ^1^
*Hericium* plv. tit. 5% polysaccharides	262.5 mg	-	525 mg	-
*Hericium* e.s. tit. 30% polysaccharides	112.5 μg	-	225 mg	-
Quercetin 98%	37.5 mg	-	75 mg	-
Biotin	112.5 μg	225	225 μg	450
Niacin	13.5 mg	84.3	27 mg	168.6
Berberis e.s. tit. 97% berberine	37.5 mg	-	75 mg	-
Sodium butyrate	150 mg	-	300 mg	-
*Lactobacillus acidophilus* SGL11	2.5 bn CFU ^2^	-	5 bn CFU ^2^	-
*Bifidobacterium animalis* subsp. lactis SGB06	1 bn CFU ^2^	-	2 bn CFU ^2^	-
*Lactiplantibacillus plantarum* SGL07	0.5 bn CFU ^2^	-	1 bn CFU ^2^	-

Notes: ^1^ RNF: Reference nutritional values; ^2^ CFU: Colony forming units.

**Table 2:** Baseline characteristics of patients included in the retrospective analysis.

Parameter	Nutraceutical Supplementation Group (N = 389)	Control Group (N = 210)	*p*-Value ^1^
**Age**	57.3 (46.8–64.5)	57.7 (47.8–66)	0.34
**Sex**			
Males	191 (49.1%)	99 (47.1%)	0.66
**Active smoking**	137 (35.2%)	72 (34.3%)	0.85
**Alcohol consumers**	122 (31.4%)	60 (28.6%)	0.4
**Appendectomy**	113 (29%)	60 (28.6%)	0.92
**Comorbidity**			
Cardiovascular	138 (35.4%)	68 (32.3%)	0.45
Respiratory	47 (12.1%)	26 (12.4%)	0.89
Metabolic	128 (32.9%)	49 (23.3%)	**0.015**
Musculoskeletal	50 (12.9%)	23 (11%)	0.6
Neurological	26 (6.7%)	9 (4.3%)	0.27
Other	48 (12.33%)	32 (15.2%)	0.37
**Patient’s standard diet**			
Mediterranean	323 (83%)	186 (88.6%)	0.23
Vegan/Vegetarian	10 (2.6%)	6 (2.9%)	0.89
Predominantly meat-based	31 (8%)	9 (4.3%)	0.09
Predominantly fish-based	11 (2.8%)	5 (2.4%)	0.68
Gluten-free Low	4 (1%)	3 (1.4%)	0.55
FODMAP	10 (2.6%)	1 (0.5%)	0.12
**Colonoscopy indication**			
Cancer screening	147 (37.8%)	87 (41.4%)	0.56
Cancer familiar history	101 (26%)	56 (26.7%)	0.68
Lower GI bleeding	77 (19.8%)	40 (19.1%)	0.84
Other	64 (16.4%)	27 (12.8%)	0.34
**Bowel preparation**			
2 L PEG plus citrate/simethicone ^2^	119 (30.6%)	53 (25.2%)	0.66
2 L PEG plus ascorbate ^3^	25 (6.4%)	9 (4.3%)	0.12
2 L PEG plus ascorbate ^4^	124 (31.9%)	64 (30.5%)	0.85
Magnesium citrate-based ^5^	2 (0.5%)	2 (1%)	0.4
4 L PEG ^6^	119 (30.6%)	82 (39%)	0.21
**DICA score**	158 (40.6%)	88 (41.9%)	0.1
1	98 (25.2%)	66 (31.4%)	0.55
2	41 (10.5%)	18 (8.5%)	0.66
3	19 (4.8%)	4 (1.9%)	0.08
**BBPS**	7 (6–9)	7 (6–8)	**<0.001**
**BBPS < 6 (Suboptimal)**	33 (8.4%)	32 (15.2%)	**<0.001**
**Diet before bowel preparation** (days)	3 (3–4)	3 (3–3)	**<0.001**

Notes: Continuous variables are expressed as median (interquartile range), while categorical/ordinal variables are presented as frequency (percentage relative to the total). ^1^ The two-tailed *p*-value, with a significance level set at a 5% alpha error, was calculated to determine whether a significant difference existed between the two study groups for the parameter of interest. Various bowel preparation regimens were assessed: ^2^ Clensia^®^, ^3^ Plenvu^®^, ^4^ Moviprep^®^, ^5^ Citrafleet^®^, and ^6^ SELG-ESSE^®^. FODMAP: Fermentable Oligosaccharides, Disaccharides, Monosaccharides, and Polyols; DICA: Diverticular Inflammation and Complication Assessment; BBPS: Boston Bowel Preparation Scale.

## Data Availability

The original contributions presented in the study are included in the article, further inquiries can be directed to the corresponding author.

## Supplementary Materials

The following supporting information can be downloaded at: https://www.mdpi.com/article/10.3390/nu17193152/s1, Table S1: Detailed characteristics of probiotic *Lactobacillus acidophilus* SGL11; Table S2: Detailed characteristics of probiotic *Bifidobacterium animalis subsp. lactis* SGB06; Table S3: Detailed characteristics of probiotic *Lactiplantibacillus plantarum* SGL07.

## Appendix A

^‡^ The HERICIUM-COLON Study Group is listed in Table A1 below, and the names are presented in alphabetical order.

**Table A1:** The HERICIUM-COLON study group.

Name and Surname	Affiliation
**Luciana Avallone**	Dipartimento di Medicina, Endoscopia Digestiva e Malattie Apparato Digerente, AUSL Ferrara, Ospedale del Delta, Ferrara, Italy
**Stefania Baroni**	Private practice, Milano, Italy
**Barbara Battista**	UOC di Gastroenterologia ed Endoscopia Digestiva Ospedale Santa Scolastica di Cassino, Roma, Italy
**Antonio Bordonaro**	UOS Gastroenterologia ed Endoscopia Digestiva, Ospedale Umberto I, Enna, Italy
**Filippo Bova**	Gastroenterologia, Ospedale Metropolitano di Reggio Calabria, Italy
**Antongiulio Bucci**	UOC di Gastroenterologia ed Endoscopia Digestiva, Ospedale San Paolo, Bari, Italy
**Monica Carta**	Gastroenterologia, Ospedale SS Annunziata, Sassari, Italy
**Carlo Cellini**	UOC di Gastroenterologia ed Endoscopia Digestiva, Ospedale Mazzini, Teramo, Italy
**Andrea Cocco**	UOC Transmurale Territoriale Gastroenterologia ed Endoscopia Digestiva Ospedale Sandro Pertini, Roma, Italy
**Berardino D’Ascoli**	UOSD Gastroenterologia, Ospedale Madonna delle Grazie, Matera, Italy
**Francesco Decembrino**	UOC Gastroenterologia ed Endoscopia Digestiva, E.E. Ospedale Regionale F. Miulli, Acquaviva delle Fonti, Bari, Italy
**Massimo Devani**	Reparto Endoscopia Digestiva Ospedale Rho, Milano, Italy
**Vincenzo Di Martino**	UOSD Endoscopia Digestiva-Ospedale dei Colli CTO, Napoli, Italy
**Domenico Dominjanni**	Ambulatorio Territoriale di Gastroenterologia ASL RM3, Roma, Italy
**Enrica Evangelista**	Ambulatorio Territoriale Gastroenterologia ASL Provincia di Frosinone, Frosinone, Italy
**Lucia Falconi**	UOC Gastroenterologia ed Endoscopia Digestiva, Ospedale Misericordia, Grosseto, Italy
**Emanuele Ferrante**	UO di Endoscopia Digestiva, ASL Napoli II Nord, PO San Giuliano, Giugliano in Campania, Italy
**Katia Feole**	Servizio di Gastroenterologia ed Endoscopia Digestiva Policlinico Casilino, Roma, Italy
**Roberto Faggiani**	UOC Gastroenterologia ed Endoscopia Digestiva Azienda Ospedale San Camillo Forlanini, Roma, Italy
**Maria Domenica Franzese**	UOC Gastroenterologia ed Endoscopia Digestiva, Ospedale Santa Maria delle Grazie, Pozzuoli, Italy
**Maurizio Giovannone**	UOC Gastroenterologia ed Endoscopia Digestiva Azienda Ospedale San Camillo Forlanini Roma, Italy
**Donato Iannuzziello**	Unità di Gastroenterologia, Mater Dei Hospital, Bari, Italy
**Nicola Ierfone**	UOC Chirurgia e Diagnostica Endoscopica-P.O. S.S. Filippo e Nicola, Avezzano, Italy
**Giampiero Indennitate**	Servizio di Endoscopia Digestiva, Casa di Cura Ulivella e Glicini, Firenze, Italy
**Luca Isola**	Gastroenterologia, Ospedale Evangelico Internazionale Genova Voltri, Genova, Italy
**Roberto Lamanda**	UOC Gastroenterologia ed Endoscopia Digestiva, Ospedale Santa Maria delle Grazie, Pozzuoli, Italy
**Maria Antonia Lai**	Endoscopia, Ospedale Businco, Azienda Ospedaliera Brotzu, Cagliari, Italy
**Giovanni Lombardi**	UOC Gastroenterologia ed Endoscopia Digestiva, Ospedale Cardarelli, Napoli, Italy
**Tammaro Maisto**	Servizio di Endoscopia Digestiva, Ospedale Santa Maria della Pietà, Casoria, Italy
**Attilio Mancino**	Private practice, Marsala, Italy
**Giampiero Manes**	Reparto Endoscopia Digestiva Ospedale Rho-Garbagnate, Garbagnate, Italy
**Francesca Menasci**	Servizio di Gastroenterologia ed Endoscopia Digestiva Policlinico Casilino, Roma, Italy
**Giammarco Mocci**	Gastroenterologia, Ospedale Brotzu, Cagliari, Italy
**Antonino Morabito**	Endoscopia Digestiva, Presidio Ospedaliero di Tropea, ASP Vibo Valentia, Italy
**Caterina Mucherino**	UOC Gastroenterologia AORN Sant’Anna e San Sebastiano, Caserta, Italy
**Domenico Napoletano**	Servizio di Endoscopia Digestiva, Clinica San Paolo, Aversa, Italy
**Nicoletta Orzes**	Private practice, Gorizia, Udine, Italy
**Giuseppe Parisi**	Private practice, Sant’Agata di Militello (ME), Italy
**Marta Patturelli**	UOC Gastroenterologia ed Endoscopia Digestiva, Ospedale Cardarelli, Napoli, Italy
**Giuseppe Pianese**	UOS Endoscopia Digestiva UOC Gastroenterologia Universitaria Ospedale Santa Maria Goretti, Latina, Italy
**Giacomo Rando**	UOC Gastroenterologia ed Endoscopia Digestiva-Ospedale Spirito Santo, Pescara, Italy
**Rocco Ranaldo**	UOC Medicina Interna, Ospedale Mazzolani Vandini, AUSL Ferrara, Italy
**Raffaella Reati**	Reparto Endoscopia Digestiva Ospedale Garbagnate, Milano, Italy
**Antonio Romano**	Private practice, Pisa, Italy
**Stefano Rodinò**	Azienda Ospedaliero Universitaria Renato Dulbecco, Catanzaro, Italy
**Antonella Scarcelli**	UOC Gastroenterologia ed Endoscopia Digestiva-AST Pesaro e Urbino, Pesaro/Fano, Italy
**Silvia Sedda**	UOC Gastroenterologia ed Endoscopia Digestiva, Ospedale del Mare, Napoli, Italy
**Luca Sediari**	Gastroenterologia, Ospedale Santa Maria della Misericordia, Perugia, Italy
**Corrado Selvaggio**	Gastroenterologia, Ospedale Maggiore di Modica, Ragusa, Italy
**Nicola Sinnona**	Servizio Gastroenterologia ed Endoscopia Digestiva Clinica San Marco, Latina, Italy
**Francesco Tedone**	UOC Gastroenterologia ed Endoscopia Digestiva, Ospedale Misericordia, Grosseto, Italy
**Giuseppe Tarantino**	AUO delle Marche-Clinica di Gastroenterologia, Epatologia ed Endoscopia Digestiva, Ancona, Italy
**Lorenza Tifi**	Gastroenterologia, USL Umbria 1, Perugia, Italy
**Elisa Tiratterra**	Gastroenterologo, Villa Erbosa-Gruppo San Donato, Bologna, Italy
**Giuliana Vespere**	UOC Gastroenterologia ed Endoscopia Digestiva, Ospedale del Mare, Napoli, Italy
**Gabriele Vigliotti**	UOSD Endoscopia Digestiva, Ospedale dei Colli CTO, Napoli, Italy
**Paolo Usai Satta**	Gastroenterologia, Azienda Ospedaliera Brotzu, Cagliari, Italy
**Rita Ortenzi**	USL Umbria 1, Perugia, Italy
