# Supplementary material for: Management of Post-Colonoscopy Syndrome with a Nutraceutical Intervention Based on Hericium erinaceus: A Retrospective Two-Arm Multicentre Analysis
Source: Nutrients. 2025 Oct 2;17(19):3152. doi: 10.3390/nu17193152 (PMC12526327; doi:10.3390/nu17193152)
Supplement: Supplementary file 1 [file nutrients-17-03152-s001.zip › nutrients-3870156-supplementary.pdf]

Table S1. Detailed characteristics of probiotic *Lactobacillus acidophilus* SGL11

Parameter	Data
**Product name**	*Lactobacillus acidophilus SGL11 >100 Mld UFC/gr*
**Description**	Freez-dried lactic ferments color white/ cream, with characterisitic odor
**Appearance**	Spherical microparticles
**Loss on drying**	< 10.0 % (1 h, 105°C)
**pH (solution 1:10)**	5.0 ± 1.5
**Particle size**	60 Mesh (250 micron) 5-20%; 120 Mesh (125 micron) 25-50%; >120 Mesh 40-70%
**Carrier**	Maltodextrin from maize
**Live cells counted (met. ISTISAN 08/36 – Proc. Int. 007)**	> 100 MLD CFU/g
**Heavy metals**	Pb < 3 ppm, Cd < 1 ppm, Hg < 0.1 ppm
**Enterobacteriaceae**	≤ 10 CFU/g
**Yeast and Moulds (TYMC)**	≤ 100 CFU/g
**Pathogens**	E. coli: absent/ 1 g; Staphylococcus A.: absent/1 g; Listeria spp: absent/25 g
**Optimal processing conditions**	Environmental humidity <30%U.R , Temperature 15°C /-5°C; closing of the packaging in the controlled environment
**Storage for closed package**	Stored at temperature less than 4 °C or freezed (-18/-25°C)

Table S2. Detailed characteristics of probiotic *Bifidobacterium animalis subsp. lactis* SGB06

Parameter	Data
**Product name**	B. animalis ssp Lactis SGB06 > 50 Billion / B. animalis ssp Lactis SGB06 > 50 Billion
**Description**	Freez-dried lactic ferments color white/ cream, with characterisitic odor
**Appearance**	Spherical microparticles
**Loss on drying**	< 10.0 % (1 h, 105°C)
**pH (solution 1:10)**	6.5 ± 1.5
**Particle size**	60 Mesh (250 micron) 5-25%; 120 Mesh (125 micron) 30-45%; >120 Mesh 40-70%
**Carrier**	Maltodextrin from maize
**Live cells counted (met. ISTISAN 08/36 – Proc. Int. 007)**	> 50 MLD CFU/g
**Heavy metals**	Pb < 3 ppm, Cd < 1 ppm, Hg < 0.1 ppm
**Enterobacteriaceae**	≤ 10 CFU/g
**Yeast and Moulds (TYMC)**	≤ 100 CFU/g
**Pathogens**	E. coli: absent/ 1 g; Staphylococcus A.: absent/1 g; Listeria spp: absent/25 g
**Optimal processing conditions**	Environmental humidity <30%U.R , Temperature 15°C /-5°C; closing of the packaging in the controlled environment
**Storage for closed package**	Stored at temperature less than 4 °C or freezed (-18/-25°C)

Table S3. Detailed characteristics of probiotic *Lactiplantibacillus plantarum* SGL07

Parameter	Data
**Product name**	Lactobacillus plantarum SGL07 > 100 Mld UFC/g
**Description**	Freez-dried lactic ferments color white/ cream, with characterisitic odor
**Appearance**	Spherical microparticles
**Loss on drying**	< 10.0 % (1 h, 105°C)
**pH (solution 1:10)**	5.5 ± 1.5
**Particle size**	60 Mesh (250 micron) 5-20%; 120 Mesh (125 micron) 25-30%; >120 Mesh 50-70%
**Carrier**	Maltodextrin from maize
**Live cells counted (met. ISTISAN 08/36 – Proc. Int. 007)**	> 100 MLD CFU/g
**Heavy metals**	Pb < 3 ppm, Cd < 1 ppm, Hg < 0.1 ppm
**Enterobacteriaceae**	≤ 10 CFU/g
**Yeast and Moulds (TYMC)**	≤ 100 CFU/g
**Pathogens**	E. coli: absent/ 1 g; Staphylococcus A.: absent/1 g; Listeria spp: absent/25 g
**Optimal processing conditions**	Environmental humidity <30%U.R , Temperature 15°C /-5°C; closing of the packaging in the controlled environment
**Storage for closed package**	Stored at temperature less than 4 °C or freezed (-18/-25°C)
